# Exploring physical literacy in children aged 8 to 12 years old: a cross-cultural comparison between China and Greece

**DOI:** 10.1186/s12889-022-14507-9

**Published:** 2022-11-17

**Authors:** Ming Hui Li, Vasiliki Kaioglou, Rui Si Ma, Siu Ming Choi, Fotini Venetsanou, Raymond Kim Wai Sum

**Affiliations:** 1 Department of Sports Science and Physical Education, The Chinese University of Hong Kong, Hong Kong SAR, China; 2 School of Physical Education and Sport Science, National and Kapodistrian University of Athens, Athens, Greece; 3 School of Physical Education, Jinan University, Guangzhou, China; 4 Physical Education Unit, The Chinese University of Hong Kong, Hong Kong SAR, China

**Keywords:** CAPL-2, China, Cross-cultural comparison, Greece, Physical literacy

## Abstract

**Background:**

The concept of physical literacy (PL) has been advocated as a crucial determinant for increasing the quality and quantity of movement and physical activity (PA). Children’s PL has been rarely compared across countries, although it has shown low levels in many countries. This study aimed to explore and compare children’s PL from China and Greece.

**Methods:**

A total of 327 Chinese (47.1% boys) and 295 Greek children (48.1% boys) aged 8 to 12 years participated in this study. Children’s PL was objectively assessed by the Chinese and Greek version of the Canadian Assessment of Physical Literacy, 2nd edition, which consists of four domains: Daily Behavior, Physical Competence, Motivation and Confidence, and Knowledge.

and Understanding. Univariate analysis of covariance (ANCOVA) and multivariate analysis of covariance (MANCOVA) on total PL and domain scores were conducted in both countries, respectively.

**Results:**

MANOVA revealed significant differences in total PL and distribution scores between two countries (Pillais’ trace = 0.260, F = 53.855, *p* < 0.001, η^2^ = 0.260), with Greek children scoring better than Chinese. Nevertheless, most children failed to present an adequate PL level as they were mostly in the “progressing” stage. The chi-square denoted that the distribution of Chinese and Greek participants across the CAPL-2 interpretive categories was statistically different for total PL (χ^2^ [3] = 18.726, *p* < 0.001, Cramer’s V = 0.174), with more Greek children being classified as “achieving” and “excelling”.

**Conclusions:**

The variance between Chinese and Greek children may be attributed to cultural differences in the context of PA, such as PE policies and settings. The relatively low level of PL shown in both countries echoes the global trend of the declining PA among children, with an increasing amount of their time spent in a sedentary lifestyle. These findings highlight the need to consider children’s cultural factors and pedagogical strategies in terms of developing their PL. Future research is required to explore the impact of cultural background on PL development among children and appropriate strategies to migrate their influence.

## Background

The benefits of physical activity (PA) for children are garnering more attention worldwide as decreasing PA and increasing recreational screen time are among the major global health issues and are responsible for increased risk of childhood obesity, high blood pressure, and mortality [1]. In many countries, efforts have been devoted to promoting PA and developing a better understanding of, and potential solutions for, health and wellbeing during childhood and adolescence [2]. The update of the WHO recommendations on PA have provided evidence for worldwide children and adolescents aged 5 to under 18 years old to accumulate at least 60 min of moderate/vigorous-intensity PA (MVPA) per day to maintain good physical and mental health and well-being [3]. There are 25 European countries in collaboration to make effort by analyzing data within obesity surveillance initiate to promote regular PA as a preventative measure to help reduce a wide variety of health risk factors across all ages, genders, ethnicities, and socioeconomic subgroups [4]. However, research has shown that more than half of 6-to-11-year-old children do not fulfill the recommended level of PA [5], and this unhealthy lifestyle acquired during childhood can be tracked into adolescence and adulthood [6]. In response to this situation, the multidimensional construct of physical literacy (PL) has been recognized as an important prerequisite to PA and sports [7, 8] because of its potential to facilitate health-related, whole-person development, especially when the formative aspects are laid in early childhood [9]. The concept of PL is regarded as a disposition that should be nurtured throughout all phases of life [10]. This notion describes the interface of several constructs in relation to children and adolescents’ PA participation, which encompasses the motivation, confidence, physical competence, and knowledge required to value and take responsibility for life [11]. PL is a critical worldwide public health initiative that supports ongoing engagement with PA [12] and is deemed the ultimate outcome in the domain of physical education (PE) [13]. PL is rapidly becoming the guiding ideology in promoting PE reforms in school settings to obtain maximum health benefits [14].

As the level of PL among children has been reported differently by various countries, it could be influenced by the cultural context, as well as the social and physical environment wherein the children are brought up [15–17]. Thus, a better understanding of such differences in PL may contribute to more efficient interventions with regard to children’s health and well-being. In Canada’s 2016 report card, PL was given a D+ grade, inferring that 36% of 8- to 12-year-olds children in Canada could meet or exceed the minimum level recommended for PL and 39% of them have met the PA recommendation [18]. For children in Australia, less than 20% achieved the requirement, depicting the low level of PL in this country [19]. Although the actual PL levels could not be achieved in their report regarding Australian children, research shows that certain groups of youth with low socioeconomic status, such as those from economically disadvantaged or cultural backgrounds, may be even more at risk of low PL or its correlates, such as fundamental movement skills (FMS) [20]. A previous study outlined that cultural factors may significantly affect the FMS-correlates in the context among Asian-speaking and English-European children aged 9 to 11 years [20], while the difference is mainly reflected by object control competence. Studies also found that cultural background may also influence the correlates of fitness levels between two Asian countries [21], and motor competence between two neighboring countries situated within the same continent [22]. Hence, it is necessary to focus on cross-country comparisons when adopting assessment tools, wherein the possible differences in children’s PL may provide a better understanding of different cultural backgrounds for PL development.

Canadian Assessment of Physical Literacy (CAPL) is the first valid and reliable protocol, supported by empirical evidence, used to monitor children’s PL in Canada [23]. Through a thorough curriculum review and a 3-round Delphi process, CAPL underwent extensive consultations with practitioners and researchers in the specific field, who confirmed the applicability of the final model [24]. Although high examiner and participant burden was conveyed by CAPL participants [25], a recently refined CAPL model, which is known as the second edition of CAPL (CAPL-2), was developed according to the recommendations of experts during the Delphi process [24, 26]. This version is much more streamlined, adhering appropriately to the most prominent definition of PL for applied use among children between the ages of 8 to 12 years, and consists of four domains: physical competence, daily behavior, knowledge and understanding, and motivation and confidence, accumulating a total score of 100 points. CAPL has been applied in many countries as a comprehensive assessment protocol, either as a whole or in part [15, 27–31]. A category interpretation is presented for each participant to distinguish his/her progressive score, namely “beginning”, “progressing”, “achieving”, and “excelling” [23].

Although widely used to ascertain children’s PL level [30, 32], relevant knowledge for understanding the discrepancy of PL between countries is still very limited. For example, few studies [15, 17] have explored how PL has been developed among boys and girls at different ages or in varying cultural and pedagogical contexts wherein sex differences may impact the level of PL between different countries/districts. Both studies distinguished the performance of boys to be better than that of girls in the combination test of motor skills assessment, while the scores may vary in the overall performance score between the two studies (i.e. Greek children performed lower than their Canadian peers [15]. Sex differences could be examined in these populations to elucidate their potential influence on children’s PL development. Additionally, cross-cultural research can offer valuable insights into how different levels of PL have been developed in distinct cultural contexts and how these tests, measuring specific domains of PL, are sensitive to cultural differences. Concerning the significance and rationale behind this notion, there is a dearth of research comparing children’s PL levels between countries.

This study provides a comparison of PL levels between children belonging to two countries - Greece and China - located on two different continents (i.e. Europe and Asia, respectively). The children may share some discrepancies across the continents. Greece is a small country in the Mediterranean region, where 85% of children have been reported participating in low PA levels [15, 33]. Similarly, the Hong Kong Report Card - an evidence-based synthesis on PA behaviors in children and youth - specified that over 90% of Chinese school-aged children and youth present have inadequate participation in PA [34]. Such findings might be further explored through a comparison of PL levels, especially when it is attributed to cross-cultural differences in educational systems and PE policies across various countries [35]. In this case, streamlining assessment and international collaborations while exploring PL is of vital importance, as it may present benefits in terms of better understanding on a global level about how physically literate the children are and what cultural factors help to better facilitate the development of PL.

Therefore, this study aimed to 1) examine the PL level of 8- to 12-year-old children from Greece and China using CAPL-2; 2) compare the score distribution of both samples across the interpretative categories. Based on previous research on the PL level of children [15–17], the PL level of Chinese and Greek children was hypothesized to be similarly low in the overall findings, but some differences were presumed to exist with regard to the cultural background (within the two countries) and sex.

## Methods

### Participants

Participants were 327 Chinese (47.1% boys) and 295 Greek (48.1% boys) children aged 8 to 12 years. In both countries, a convenience sampling approach was adopted. In Greece, data were collected from four primary schools in Athens, Thessaloniki, and Naousa from May 2018 to June 2019, while in China, data were collected from two primary schools in Hong Kong SAR and Shenzhen between March 2018 and September 2018. More than 85% of 8- to 12-year-old students in those schools were recruited in the study. Written informed consent was acquired from the parents or guardians of each participant. Before data collection, participants were asked to give their verbal consent as well. This study was approved by the University Survey and Behavioral Ethics Committees in both countries.

### Measures

Demographic information was collected from both countries using questionnaires. All four domains of the CAPL-2 were assessed in accordance with the CAPL-2 manual (available at www.capl-eclp.ca/international/) to measure the level of PL. First, the daily behavior domain included two components: objectively measured step counts (25 points) and self-reported moderate to vigorous physical activity (MVPA; 5 points), defined as the number of days per week a child engages in activities that make them breathe harder and faster. Step counts of the Greek children were measured using pedometers, and ActiGraph GT3X+ accelerometers were utilized in the case of the Chinese children. Both were positioned over the hip bone on the right-hand side of their body and had to be worn for seven consecutive days, excluding the time when the children engaged in water activities, bathing and sleeping. According to the CAPL-2 manual, some discrepancies existed between the accelerometer-pedometer-determined steps, with only a small difference in correlation present when research adhered to known sensitivity thresholds [16]. Thus, the valid wear time for data deduction was determined to be at least 10 h/day for a minimum of four days [36]. The total score for this domain was 30 points.

The physical competence domain included three measures: a) FitnessGram 15 m/20 m Progressive Aerobic Cardiovascular Endurance Run (PACER) [37] to evaluate aerobic fitness; b) Plank Assessment of Torso Strength [38] for testing musculoskeletal endurance; and c) the Canadian Agility and Movement Skill Assessment (CAMSA) [39], a sequence test combining fundamental, complex and combined movement skills, such as catching, throwing, skipping and hopping to assess motor competence. Further information about scoring and CAMSA implementation is detailed in the data collection procedures. The maximum score for physical competence was 30 points, with each assessment representing 10 points.

The knowledge and understanding domain assessed a child’s knowledge regarding PA through five questions that equaled 10 points [40]. The motivation and confidence domain evaluated a child’s confidence in their ability to be physically active and their motivation to participate in PA. This notion was defined via four aspects: predilection, adequacy, intrinsic motivation, and PA competence (three items in each aspect). The total motivation and confidence domain score equaled 30 points.

The use of CAPL-2 to measure the PL level of Chinese and Greek children is valid since this tool has been cross-culturally adapted and validated in both China [16] and Greece [29], with the participation of 327 and 576 children, respectively.

### Data collection procedures

In both China and Greece, a group of at least ten experienced appraisers was used to administer the assessment procedure. All the appraisers completed a training course to ensure consistent scoring and implementation. Data were collected at school sites during two scheduled PE lessons, with the presence of at least 5 appraisers each time (both male and female). At the first school visit, the participants completed the CAMSA and plank protocols, along with the knowledge and understanding questionnaire, while at the second visit they were assessed in the PACER and the motivation and confidence questionnaire. The assessments were administered according to specific instructions included in the CAPL-2 manual to ensure equal treatment of all participants. No additional school visits were scheduled for children who were absent or unable to participate due to injury or refusal.

### Data analysis

At first, data cleaning procedures were applied. Data were analyzed using IBM SPSS Statistics for Windows, version 25.0. According to the main research questions, for the investigation of potential differences between the two countries (i.e., China and Greece) and sexes, the following analyses were conducted: a) a 2 × 2 univariate analysis of covariance (ANCOVA) on the total PL score; b) a 2 × 2 multivariate analysis of covariance (MANCOVA) on the four CAPL-2 domain scores; and c) separate 2 × 2 ANCOVAs on each measure score. In all the above analyses, age was employed as a covariate because children’s age has been found to have a positive effect on total PL and CAPL-2 domain scores [15, 17]. Based on the information from the CAPL-2 manual, participants from both countries were classified across the CAPL-2 interpretive categories for total PL and CAPL-2 domains. The proportion (%) of each category for the above measures was also calculated. Moreover, a chi-square (χ^2^) test was applied to compare the distributions of the Chinese and Greek children across the CAPL-2 interpretive categories for the total PL and CAPL-2 domains.

The significance level for all analyses was set at *p* < 0.05. For the interpretation of the results of univariate/multivariate analyses, the reported effect sizes (η^2^) were taken into consideration (η^2^ > 0.14 refers to practical importance [41]. Likewise, the chi-square results were interpreted considering the respective effect sizes (i.e., Cramer’s V). Cramer’s V is classified as low (Cramer’s V = 0.1–0.2), moderate (Cramer’s V = 0.2–0.4), relatively strong (Cramer’s V = 0.4–0.6) and strong (Cramer’s V > 0.6)] [42].

## Results

The estimated marginal means and standard errors for total PL, CAPL-2 domains, and individual measure scores stratified by country and sex, are summarized in Table 1. The results of univariate and multivariate analyses are displayed in Table 2.

**Table 1:** Marginal means, standard errors for total PL, CAPL-2 domain and individual measure scores by country and sex

	China			Greece		
	Boys (*n* = 154)	Girls (*n* = 173)	Total (*n* = 327)	Boys (*n* = 142)	Girls (*n* = 153)	Total (*n* = 295)
Daily Behaviour						
Domain score (0–30)	13.1 ± 0.4	10.3 ± 0.4	11.8 ± 0.3	10.8 ± 0.4	10.1 ± 0.4	10.5 ± 0.3
Self-perceived MVPA (days/week)	4.2 ± 0.2	3.8 ± 0.2	4.0 ± 0.1	4.7 ± 0.2	4.6 ± 0.2	4.7 ± 0.1
Daily PA (steps)	8756.2 ± 230.3	7506.2 ± 218.1	8131.2 ± 159	7078.3 ± 240	6718.7 ± 231.9	6898.5 ± 167
Physical Competence						
Domain score (0–30)	17.9 ± 0.4	17.3 ± 0.4	17.6 ± 0.3	17.9 ± 0.4	16.5 ± 0.4	17.2 ± 0.3
CAMSA combined score (0–28)	21.7 ± 0.3	20.4 ± .3	21.1 ± .2	19.1 ± .3	18.2 ± .3	18.7 ± .2
20 m PACER (laps)	23.1 ± 0.9	20.7 ± .8	21.9 ± .6	28.0 ± .9	21.9 ± .9	25.0 ± .6
Plank (sec)	74.3 ± 3.6	78.7 ± 3.4	76.5 ± 2.4	87.8 ± 3.7	82.2 ± 3.6	85.0 ± 2.6
Motivation & Confidence						
Domain score (0–30)	22.5 ± 0.3	21.8 ± .3	22.2 ± .2	26.7 ± .3	26.1 ± 0.3	26.4 ± 0.2
Adequacy (0–7.5)	5.7 ± 0.1	5.6 ± 0.1	5.6 ± 0.1	6.7 ± 0.1	6.6 ± 0.1	6.7 ± 0.1
Predilection (0–7.5)	5.6 ± 0.1	5.4 ± 0.1	5.5 ± 0.1	6.8 ± 0.1	6.8 ± 0.1	6.8 ± 0.1
Intrinsic motivation (0–7.5)	6.0 ± 0.1	5.5 ± 0.1	5.8 ± 0.1	6.9 ± 0.1	6.8 ± 0.1	6.8 ± 0.1
PA competence (0–7.5)	5.6 ± 0.1	5.1 ± 0.1	5.3 ± 0.1	6.3 ± 0.1	6.0 ± 0.1	6.2 ± 0.1
Knowledge & Understanding						
Domain score (0–10)	5.6 ± 0.2	5.9 ± 0.2	5.7 ± 0.2	5.8 ± 0.2	6.1 ± 0.2	5.9 ± 0.2
Total PL score (0–100)	59.1 ± 0.9	55.3 ± 0.8	57.2 ± 0.6	61.2 ± 0.9	58.8 ± 0.9	60.0 ± 0.7

Note: *MVPA* Moderate to Vigorous Physical Activity; *CAMSA* Canadian Agility and Movement Skill Assessment; *PACER* Progressive Aerobic Cardiovascular Endurance Run

*p < .05, **p < .001

**Table 2:** F ratios, and η^2^ values for total PL, CAPL-2 domain and individual measure scores by country and sex

	Country by sex		Country		Sex	
	F	η^2^	F	η^2^	F	η^2^
Daily Behaviour						
Domain score	6.553*	0.011	8.924*	0.014	19.465**	0.031
Self-perceived MVPA (days/week)	1.486	0.002	16.697**	0.026	3.090	0.005
Daily PA (steps)	3.739	0.006	28.492**	0.044	12.267**	0.019
Physical Competence						
Domain score	0.853	0.001	1.199	0.002	5.852*	0.009
CAMSA total score	0.440	0.001	65.904**	0.097	14.392**	0.023
20 m PACER (laps)	4.575*	0.007	12.137**	0.019	23.434**	0.037
Plank (sec)	1.999	0.003	5.725*	0.009	0.028	< 0.001
Motivation and Confidence						
Domain score	0.063	< 0.001	170.571**	0.217	3.768	0.006
Adequacy	0.380	0.001	87.135**	0.124	0.922	0.001
Predilection	0.108	< 0.001	112.560**	0.154	0.435	0.001
Intrinsic motivation	2.808	0.005	81.634**	0.117	6.498*	0.010
PA competence	0.269	< 0.001	53.008**	0.079	13.662**	0.022
Knowledge and Understanding						
Domain score	0.052	< 0.001	1.159	0.002	3.259	0.005
Total PL score	0.644	0.001	10.590**	0.017	12.978**	0.021

Note. *MVPA* Moderate to Vigorous Physical Activity; *CAMSA* Canadian Agility and Movement Skill Assessment; *PACER* Progressive Aerobic Cardiovascular Endurance Run

*p < .05, **p < .001

### Differences in total PL and domain scores between Chinese and Greek children

As shown in Table 2 regarding the ANCOVA for total PL score, it was revealed that the interaction between countries and sex was not statistically significant after controlling for the effect of age (F = 25.291, *p* < 0.001, η^2^ = 0.039). For the main effects, they were significant but not practically important, with Greek participants achieving higher scores than Chinese, and boys overall performing better than girls in both countries.

Moreover, the MANCOVA applied to the CAPL-2 domain scores denoted that there were significant effects for both countries (Pillais’ trace = 0.260, F = 53.855, *p* < 0.001, η^2^ = 0.260) and sex (Pillais’ trace = 0.043, F = 6.966, p < 0.001, η^2^ = 0.043) after age (Pillais’ trace = 0.143, p < 0.001, F = 25.699, η^2^ = 0.143) was controlled. The interaction between country and sex was not statistically significant (Pillais’ trace = 0.015, F = 2.291, *p* = 0.058, η^2^ = 0.015). The findings from the following ANCOVAs manifested a statistically significant interaction in one of the four CAPL-2 domains (i.e. daily behavior), implying that Chinese boys presented higher scores than all other children. Concerning the main effects, the effect of the country was statistically significant only for motivation and confidence, with Greek participants achieving higher scores compared to the Chinese. Besides, the effect of sex was significant for physical competence, with boys’ scores being higher than that of girls. Still, regarding the above significant effects, only the country’s effect on motivation and confidence was of practical importance.

In separate ANCOVAs conducted for each measure score, only one significant interaction was traced, which was concerned with one of the physical competence measures (i.e. PACER), confirming that Greek boys obtained higher scores in this measure than all other children. Regarding the main effects of those analyses, the effect of the country was statistically significant for all individual measures, with Greek participants surpassing the Chinese in most of the measures, except for daily PA and CAMSA, for which Chinese participants scored higher. Yet, the country effect was practically important only for one of the motivation and confidence measures (i.e. predilection). Notably, the effect of sex was statistically significant for daily PA, CAMSA, PACER, intrinsic motivation, and PA competence, with boys attaining higher scores than girls. Nonetheless, this effect was practically important in none of the above cases. In all the above analyses, except for the physical competence measures, the effect of age was found to be insignificant.

### The CAPL-2 interpretive categories between Chinese and Greek children

**Fig. 1:**
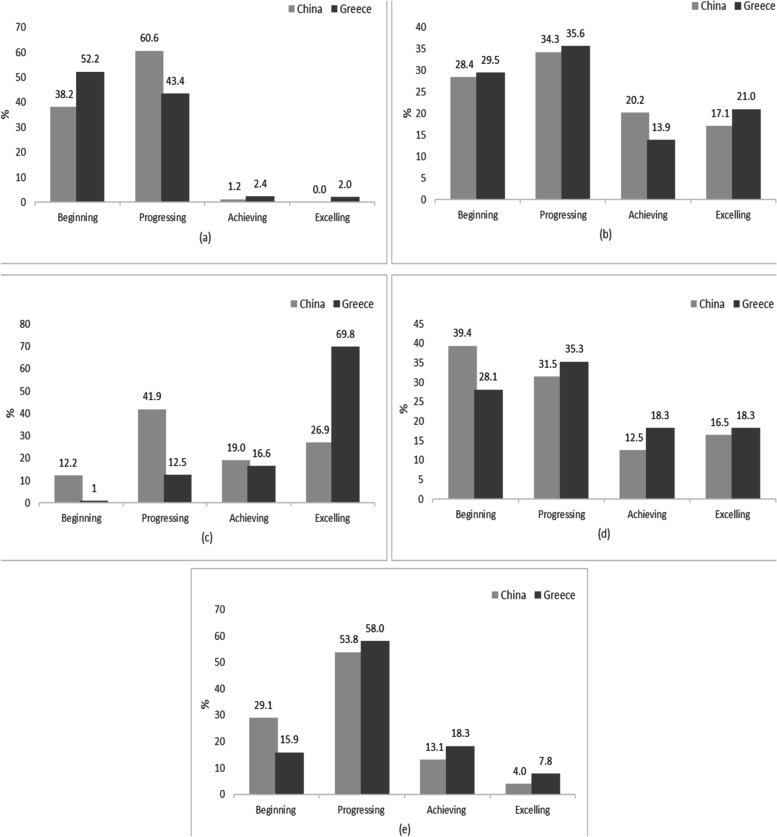
Distribution (%) of Chinese and Greek participants across the CAPL-2 interpretive categories for **a** Daily Behavior, **b** Physical Competence, **c** Motivation and Confidence, **d** Knowledge and Understanding, **e** Total PL

The distributions (in %) of Chinese and Greek participants across the CAPL-2 interpretive categories for the total PL and CAPL-2 domains are illustrated in Fig. 1. Although a higher proportion of participants from both countries was classified in the “progressing” category for the total PL domain, the proportion of Greek counterparts classified as “achieving” or “excelling” was higher compared to the Chinese. For daily behavior, a greater percentage of Greek children who participated in this study were placed in the lowest category (“beginning”), whereas a higher proportion of Chinese peers exhibited slightly greater scores and were included in the “progressing” category. For physical competence, a similar proportion of Chinese and Greek children was found in the two lower categories. For motivation and confidence, a profound disparity was apparent as most Greek children were “achieving”, whereas most of the Chinese children were “progressing”. Lastly, for knowledge and understanding, even though the performance of most of the Chinese and Greek peers fell into either the “beginning” or “progressing” category, the proportion of Greeks that were “achieving” or “excelling” was higher compared to the Chinese. The chi-square test demonstrated that the distribution of Chinese and Greek participants across the CAPL-2 interpretive categories was statistically different for total PL (χ^2^ [3] = 18.726, *p* < 0.001, Cramer’s V = 0.174), Daily Behavior (χ^2^ [3] = 23.279, p < 0.001, Cramer’s V = 0.193), Motivation and Confidence (χ^2^ [3] = 136.908, p < 0.001, Cramer’s V = 0.469) and Knowledge and Understanding (χ^2^ [3] = 10.145, *p* = 0.017, Cramer’s V = 0.128), whereas it was not significantly different for physical competence (χ^2^ [3] = 4.939, *p* = 0.176, Cramer’s V = 0.089). Still, according to the reported Cramer’s V values, small effect sizes were noticeable for the above statistically significant outcomes. The only relatively strong effect size was found for the motivation and confidence domains.

## Discussion

The objective of this study was to compare the PL levels of 8- to-12-year-old children from China and Greece using the CAPL-2, along with investigating potential differences between sexes. The main findings indicated that although there were differences in total PL and distribution scores between children of the two countries, favoring Greek children, it is unfortunate that most children, regardless of nationality, failed to present an adequate PL level as they were classified as “progressing”. Interestingly, the superiority of Greek children was primarily due to the excessive motivation and confidence they demonstrated. However, it is unclear why their motivation was not compatible with their PA participation, which was noticeably low. Conversely, Chinese children proved to be more physically active than their Greek peers; however, overall, the PA levels of most children in both countries were below the recommended levels, putting their health at risk.

Attempting to interpret the PL-related differences between the two countries, the three constraint-based models were adopted to act as a framework to illustrate the aspects whereby PL may be influenced [43]. In the early childhood educational system, more attention should be given to physical activities during school time as it is a critical phase for developing PL and facilitating children’s participation in PA [32]. Given that the sex and age distributions between Chinese and Greek children were similar, it can be hypothesized that different conditions within the PA environments, i.e., PE, recess, or extracurricular activities, in the two countries may account for the cultural differences in the CAPL-2 domains.

In China, over 90% of Chinese school-aged children and youth have insufficient participation in PA [34] and only 13.1% satisfy the PA guidelines recommended by the World Health Organization, which require at least one hour of daily MVPA [44]. In Hong Kong, schools’ PE programs are a major context wherein children’s PA engagement can be fostered [45]. For a long time, the goal of education in Hong Kong comprised five components, namely “Moral, Intelligence, Physical, Social and Atheistic” [46], which required all the students to develop comprehensively and actively during their lifespan. For primary schools, although students are compulsory to typically experience two periods of PE per week or cycle week, each amounting to less than an hour of actual instruction [46]. It was required that various types of activities should be embedded into part of the PE curriculum in both Hong Kong primary and secondary schools [47]. However, as priorities were usually given to other disciplines, insufficient time may have remained for children’s participation in PA during recess or after-school periods. Especially within the Eastern culture, parents/guardians or teachers are concerned about safety issues in relation to children’s stay in schools. Although the crisis of insufficient PA on health has caused more attention in the school’s policy-making to promote PA in school, the relatively low level of PA and PL reflected in this study may result from cultural inherent or underestimates of school PE, inhibiting children’s time spent in PA and their PL development [46].

Similarly, in Greece, relevant reports indicate insufficient levels of PA participation among preschoolers [48–50] and school-aged children [15, 33]. Structured PE in Greece is an integral part of educational practices, targeting the all-around development of children. It is typically introduced in the first grade of primary school when children are around the ages of six and seven years [51]. Students’ attendance in PE lessons (45-min lessons twice or three times per week depending on school grade) is compulsory [51], providing students with the opportunity to increase their daily PA participation and additionally develop competence, knowledge, and motivation and confidence for PA. Nevertheless, Greek schools do not incorporate structured PA “breaks” during recess or other school lessons [52]. Overall, the amount of time Greek children spent in PA during school days is inadequate and does not fulfill the PA guidelines [3]. The role of school PE is crucial considering that recreational PA opportunities are not abundant and not all children have the chance to enroll in after-school programs for economic reasons [50], even though children are highly motivated to participate in such programs and their parents/guardians approve of their participation. Notably, if Greek students had the opportunity to spend more time in PA at school, this prospect would increase their daily PA participation. Moreover, although the current Greek PE curriculum emphasizes knowledge-based concepts, such as the benefits of PA, intending to promote healthy behaviors across the entire lifespan [53], students’ awareness of such concepts may not be satisfactory as Greek PE teachers generally have low confidence in applying health-related PE instruction [54, 55]. Furthermore, as there is an established relationship between fundamental motor skills and PA participation among children [55], the poor PA experiences of Greek children have likely restrained the development of their motor skills. Considering that the above relationship is longitudinal [56, 57] and that structured PA interventions are imperative for preschoolers’ motor development [58], the absence of PE from Greek preschoolers’ curriculum has limited their ability to master their fundamental motor skills as early as possible. Overall, although the Greek educational system targets the development of the various PL elements, it seems that additional actions should be taken to boost the Greek children’s active lifestyle. Fortunately, the provision of various after-school PA programs is an alternative source of PA participation and a means to encourage PL development.

Apart from differences in PE and PA contexts between the two countries, other potential sources may also be considered for the observed PL differences between Chinese and Greek children. One possible source of variation may be the fact that data were collected within different periods. In China, data were collected within six months (including summer time), with less impact from seasonal variations; while in Greece within one year, allowing for obtaining a more complete picture of children’s PA and PL. The fact that children’s PL levels in both countries were assessed by the same tool (CAPL-2), which is not only valid and reliable for these populations but also includes assessments that were novel for all the participants, is a prerequisite to guarantee comparable results [16, 29]. Nevertheless, the administration of the assessments in each country may have allowed for some variations in the scores. However, since assessment procedures are presented in every detail in the CAPL-2 manual, and in both cases experienced examiners were used to carrying out the assessments in facilities described by the respective manual, we do not assume it would significantly contribute to the different PL levels between the Chinese and Greek children.

In reference to the administration procedures, the only deviation between the two countries was the utilization of different devices for measuring PA (ActiGraph GT3X+ accelerometers vs pedometers). In China, accelerometers were distributed among all the participants, who wore them around their waist for seven consecutive days, while Greek children wore pedometers to record their daily steps. As accelerometers may be more sensitive in recording daily steps and providing reliable information on PA intensities, research has indicated that accelerometers may provide a better picture of PA [59]. Still, previous research has confirmed the redundancy of pedometers in the presence of more sophisticated devices [23]. However, it has been reported that there is a slight discrepancy between accelerometer-pedometer-determined steps, with only a small difference in correlation when research adhered to known sensitivity thresholds [16], which we believe would not be the main reason for the differences shown in PL levels between two countries.

Having a closer look at the domain-specific differences between the two countries, the most distinct one was the excessive motivation and confidence for PA that the Greek children presented in comparison to their Chinese peers. This difference in the affective domains of PL is difficult to be justified. It can be inferred that it is due to the more effective practices adopted in the contexts of education and PA. Alternatively, it could be attributed to the inherent personality traits of Greek people (e.g., enthusiasm, eagerness, optimistic view); however, there is no empirical proof to support this notion.

At this point, it is important to highlight that despite their difference in PL scores, Chinese and Greek children did not distinctively differ from Canadian children who similarly demonstrate “progressing” PL level [15]. Generally, reports from different countries worldwide indicate that the PL level of children is not satisfactory [60, 61]. More than any cultural difference, this phenomenon could be attributed to the world trending decrease in children’s PA levels, as it is known that more than half of 6–11-year-old children do not adhere to the recommended PA guidelines [5]. With increasing age, this decline in PA participation during late childhood and adolescence should be a primary concern for non-organized PA [62], because it may influence further PL development. The lifestyles of Europe and Asia have changed over the past 40 years, mainly due to advancements in technology, a fact that resulted in how children spend their leisure time, with more time spent in sedentary behavior and therefore less PA participation [35]. Given that Cairney, Dudley [12] model highlights the bidirectional relationship between PA and PL, the downward trends of children’s PA levels may impact their overall PL levels. Taking this into account, PA and PL promotion should be addressed at the policy level across nations.

In reference to sex differences, the PL level of boys in this study was found to be higher than those of girls due to the greater physical competence level they presented. This is also evident in former studies regarding Chinese and Greek children [15, 16]. However, in some cases, it has been indicated that differences in PL between boys and girls are not practically important [15] or are negligible [63]. Overall, it seems that during this formative stage, sex differences in PL are not major, and all children regardless of sex need equal support in their PL journey.

Even though this study presents a vital cross-cultural perspective regarding PL levels of children between countries, it has several limitations. First, children’s PL levels assessed by the CAPL-2 could not be compared with other assessment tools in the same population, such as *PLAYfun* by Canadian Sport for Life and Passport for Life by PHE Canada [64]. This incomparability may lead to some differences in the interpretation of PL levels even though the CAPL was the first to provide both valid and reliable data and a comprehensive protocol to monitor children’s PL. Second, the measurement used for students’ PA in Greece and China was different (accelerometers vs pedometers), which may be seen as a reason for causing the differences in PA as the findings shown. Other differences regarding data collection between two countries should include the lack of intercoder and intracoder reliability for the questionnaires used, different time points when conducting data collections in two countries, which may have imposed potential bias in the findings, and these should be interpreted with cautions. Another limitation of this study is the uneven ratio of the sample size to the total population within the country. Compared to Greece, the sample size in China is not large enough to represent the overall PL of children in China. However, there are difficulties in acquiring a representative sample size within a nation as large as China. Therefore, future research should include more Chinese children to investigate cross-cultural differences in these PL domains to gain a better understanding of children’s PL at a global level and the current findings should be generalized with caution. Nonetheless, the utilization of a standardized and robust assessment tool that is easy to use in educational settings is the strength of this study [25]. Importantly, this research has provided a cross-cultural perspective of PL levels in a large sample of young children in two different countries.

## Conclusion

This study provides valuable information on cross-cultural comparison of PL levels among children via the CAPL-2. The outcomes verify that the Greek children scored higher on overall PL than the Chinese children. However, most children, regardless of their nationality, were not able to present an adequate PL level as they were mostly in the “progressing” stage. These findings may be due to potential differences in educational and/or PA contexts, such as PE and organized sports; however, future research is required to inspect the impact of cultural background on PL development and its correlates if a greater sample is allowed.

## Data Availability

The data generated during this study are not public because availability was not included in the study plan approved by the ethics committee and in the informed consent obtained from the participants. However, the data are available from the corresponding author on reasonable request.

## Abbreviations

CAMSA: Canadian Agility and Movement Skill Assessment
CAPL: Canadian Assessment of Physical Literacy
CAPL-2: Canadian Assessment of Physical Literacy, Second Edition
MANOVA: Multivariate analysis of variance
MVPA: Moderate-to-vigorous physical activity
PA: Physical activity
PACER: Progressive Aerobic Cardiovascular Endurance Run
PE: Physical education
PL: Physical literacy
